# A TMPRSS2‐ERG gene signature predicts prognosis of patients with prostate adenocarcinoma

**DOI:** 10.1002/ctm2.216

**Published:** 2020-12-02

**Authors:** Emily Zhou, Baoyi Zhang, Kenneth Zhu, Evelien Schaafsma, Runjun D. Kumar, Chao Cheng

**Affiliations:** ^1^ Department of Biosciences Rice University Houston Texas USA; ^2^ Department of Chemical and Biomolecular Engineering Rice University Houston Texas USA; ^3^ UT Southwestern Medical School The University of Texas Southwestern Medical Center at Dallas Dallas Texas USA; ^4^ Department of Biomedical Data Science Geisel School of Medicine at Dartmouth Lebanon New Hampshire USA; ^5^ Department of Molecular and Human Genetics Baylor College of Medicine Houston Texas USA; ^6^ Department of Medicine Baylor College of Medicine Houston Texas USA; ^7^ Dan L Duncan Comprehensive Cancer Center Baylor College of Medicine Houston Texas USA; ^8^ Institute for Clinical and Translational Research Baylor College of Medicine Houston Texas USA

Dear Editor,

The transmembrane protease serine 2‐ETS‐related gene (TMPRSS2‐ERG) fusion occurs in >50% of prostate cancers, leading to upregulation of the transcription factor ERG and tumor cell sensitivity to androgen.^1^ The fusion is associated with more aggressive manifestations of prostate cancer.^2^ Here, we developed a gene signature that recapitulated the pathway activity downstream of the TMPRSS2‐ERG fusion event and applied it to predict patient prognosis in prostate cancer.

The gene signature was defined by performing a logistic regression on every gene in The Cancer Genome Atlas prostate adenocarcinoma (TCGA‐PRAD) dataset. TMPRSS2‐ERG fusion status was used as the response variable, while gene expression level, age, and Gleason score were used as predictor variables. Based on these results, the 700 most significant genes were selected and for each of them a weight within [−1, 1] was assigned with sign indicating up‐ and downregulation, respectively. Given a new prostate cancer gene expression dataset, the weighted gene signature was applied to calculate sample‐specific scores for all samples by using a rank‐based statistic method named BASE.^3^ The resultant signature scores recapitulate the deregulated pathways downstream of the TMPRSS2‐ERG fusion event. GO enrichment analysis indicates that genes associated with hormone secretion and regulation were highly enriched in this signature (Table S3). A detailed description about the signature can be found in the Supporting Information Materials.

First, we tested whether the signature can identify tumors with TMPRSS2‐ERG fusion in the TCGA and two additional prostate cancer datasets, the Sboner data (GSE16560) and the Setlur data (GSE8402) (Table S1). We found that fusion‐positive samples exhibited significantly higher signature scores than fusion‐negative samples in all datasets (Figure 1A and Figure S1A and B). When the signature score was used to classify the two sample groups, a fairly high accuracy was achieved in all datasets as shown by the response operating characteristic curves and area under the curve scores (Figure 1B). A direct consequence of TMPRSS2‐ERG fusion is the upregulated expression of ERG. Indeed, we found that the signature score is highly correlated with the ERG mRNA level in both fusion‐positive and fusion‐negative samples (Figure S1C and D). Interestingly, a small subset of TMPRSS2‐ERG fusion‐negative samples was associated with high signature scores (Figure S1A). Of these samples, nine can be explained by the fusion of ERG with other genes such as SLC45A3. Signature scores of these samples (ERG‐Other) were lower than samples with TMPRSS2‐ERG fusions, but were significantly higher than the samples with no ERG fusion (Figure 1C). These results indicate that genomic events alternative to TMPRSS2‐ERG fusion might deregulate the same downstream pathways and thus result in similar gene expression patterns.

**FIGURE 1:**
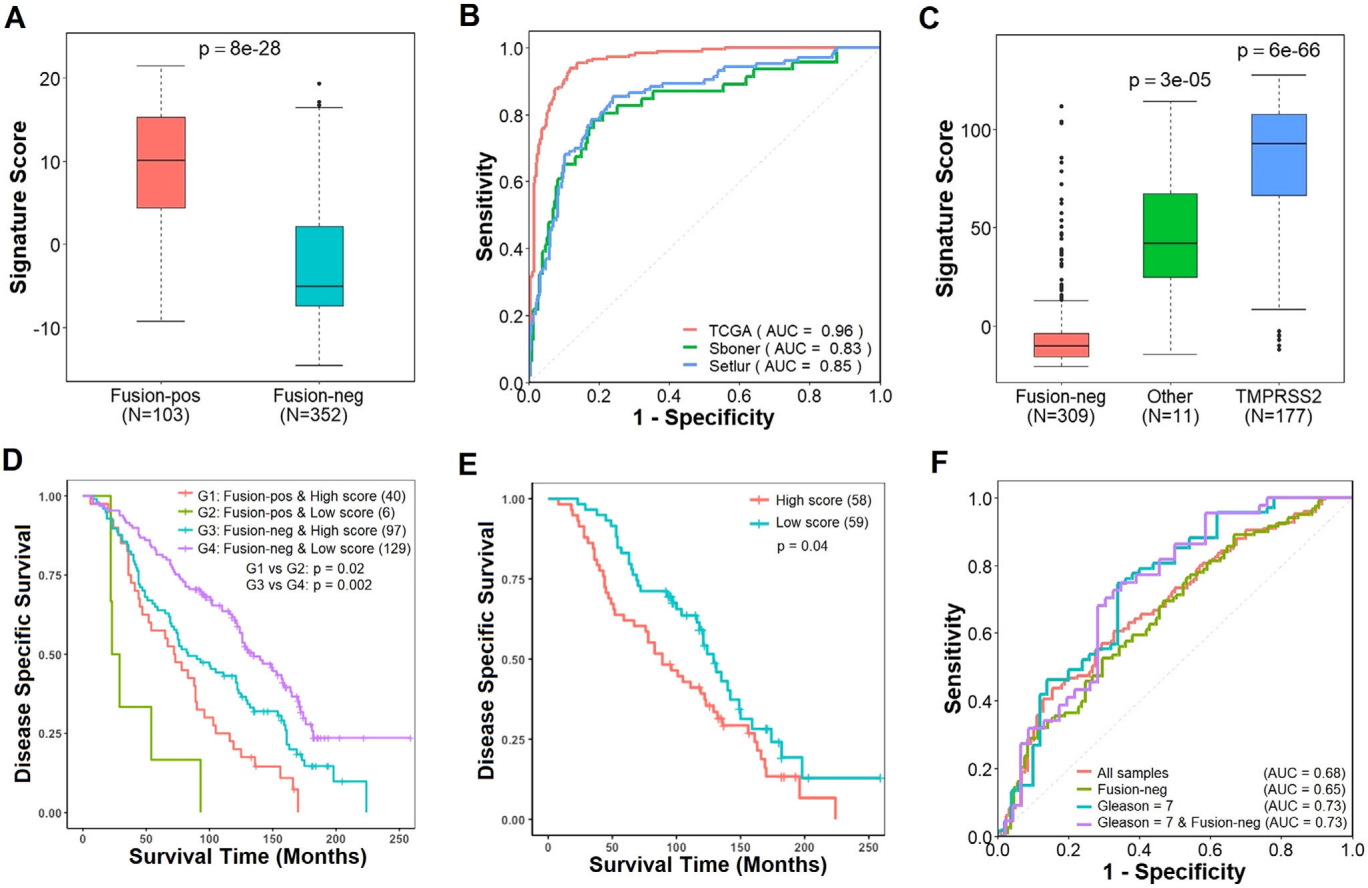
**The TMPRSS2‐ERG gene signature is predictive of patient prognosis in prostate cancer. A,** Fusion‐positive samples have significantly higher signature scores in the Setlur (GSE8402) dataset (Wilcoxon test). **B,** Signature score accurately differentiates prostate tumor samples based on fusion status in TCGA‐PRAD, Sboner (GSE16560), and Setlur (GSE8402) datasets. **C,** Samples that contain TMPRSS2‐ERG and ERG‐Other fusions have significantly higher signature scores compared to samples with no ERG fusion in TCGA‐PRAD (Wilcoxon test). **D,** Fusion‐positive samples with low signature scores exhibited significantly worse prognosis than fusion‐positive samples with high signature scores (log‐rank test). In contrast, fusion‐negative samples with high signature scores exhibited significantly poorer prognosis than fusion‐negative samples with low signature scores (log‐rank test). **E,** Patients with high signature scores exhibit significantly poorer prognosis in Gleason 7 samples (log‐rank test). **F,** Signature score differentiates indolent and lethal tumor samples with relative accuracy in all, fusion‐negative, Gleason 7, and Gleason 7 and fusion‐negative samples

Second, we examined the ability of signature score to predict patient prognosis using the Sboner data, for which disease‐specific survival information was available. We calculated the signature scores for all samples and stratified patients into two groups using the median score as the threshold. Patients with high scores have significantly poorer prognosis (*P* = 7 × 10^−05^) than those with low scores (Figure S2A). When this analysis was restricted to samples without TMPRSS2‐ERG fusion, the same result was observed: high score was associated with poor prognosis (*P* = .002, Figure 1D). Interestingly, in fusion‐positive samples high score was associated with good prognosis (*P* = .02, Figure 1D), in contrast to the negative association observed in fusion‐negative samples. Similar results but lower significance was obtained when ERG gene expression was used to stratify prostate cancer patients (Figure S2B‐D). Gleason score has been defined to categorize morphological differences and found to have high prognostic value in prostate cancer.^4^ The most common Gleason score at diagnosis^5^ and within this dataset is 7 (Figure S3A, Supporting Information Table 1), therefore we investigated the prognostic value of our signature in Gleason 7 (G7) samples. The result indicated that high score was associated with poor prognosis in all G7 (*P* = .04) and fusion‐negative G7 (*P* = .02) samples (Figure 1E, Figure S3B). In addition, signature score can accurately differentiate indolent from lethal tumor samples (Figure 1F), with lethal samples exhibiting significantly higher scores than indolent samples (Figure S3C‐F).

Finally, we examined the impact of TMPRSS2‐ERG fusion on intratumoral immune infiltration in order to understand why fusion‐positive patients have poor survival. Previous studies have reported the association of immune infiltration with cancer development and prognosis in prostate cancer.^6^ The leukocyte abundance in TCGA prostate cancer samples was obtained from the Thorsson et al's study.^7^ A comparison between TMPRSS2‐ERG fusion‐positive and fusion‐negative samples indicated significantly lower leukocyte level in the former group (Figure 2A). Then, we applied a computation method^8^ to infer the infiltration levels of six immune cell types (naïve B cell, memory B cell, CD8^+^ T cell, CD4^+^ T cell, natural killer cell, and monocyte) in all TCGA prostate cancer samples based on their gene expression profiles. Our results indicate that naive B cells (*P* = .02), natural killer cells (*P* = 2 × 10^−06^), and monocytes (*P* = 3 × 10^−07^) have significantly lower infiltration in fusion‐positive than fusion‐negative samples, while CD4^+^ T cells (*P* = 1× 10^−04^) have significantly higher infiltration in fusion‐positive samples than fusion‐negative samples (Figure 2B). These findings were further supported by significant correlation between immune infiltration and signature score (Table S2). Taken together, our results suggested that TMPRSS2‐ERG fusion is associated with reduced level of immune infiltration. Previous studies have shown that higher nonsynonymous mutation rate and lower copy number variation (CNV) in tumor samples are associated with higher immune infiltration.^9^, ^10^ We found that TMPRSS2‐ERG fusion was associated with lower nonsynonymous mutation rate (Figure 2C). However, we also observed lower level of fraction altered (Figure 2D) and lower homologous recombination deficiency scores (HRD) in fusion‐positive samples (Figure 2E). Both fraction‐altered and HRD represent CNV levels. Thereby, the immune infiltration differences between fusion‐positive and fusion‐negative samples are not due to CNV but nonsynonymous mutation rate.

**FIGURE 2:**
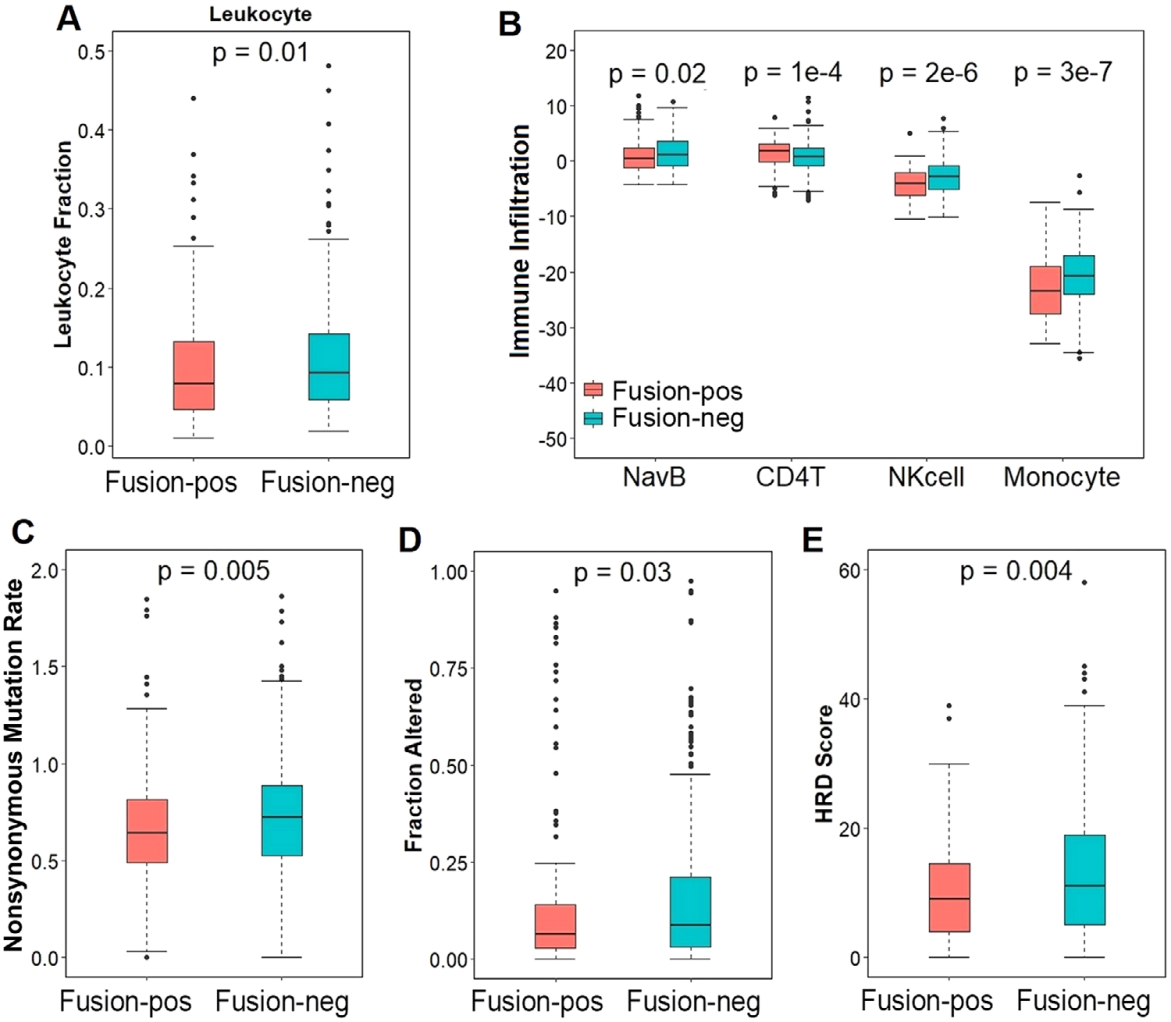
**The TMPRSS2‐ERG gene signature is associated with immune infiltration. A,** Leukocyte abundance was significantly higher in fusion‐negative samples. The number of samples in each group is found within the parenthesis. **B,** Fusion‐positive samples have significantly lower Naïve B cell (NavB), natural killer cell (NKcell), and monocyte infiltration, but lower CD4^+^ T cell (CD4T). **C,** Fusion‐positive samples have significantly lower tumor mutation burden, represented as nonsynonymous mutation rate. **D,** Fusion‐positive samples have significantly lower copy number variation burden, represented as fraction of altered genome (fraction altered). **E,** Fusion‐positive samples have significantly lower homologous recombination deficiency (HRD) scores. The *P*‐values were calculated from the Wilcoxon rank‐sum test

In summary, we defined a novel gene signature for TMPRSS2‐ERG fusion with great clinical value. The gene signature recapitulates the deregulated pathway downstream of the TMPRSS2‐ERG fusion event and is predictive of patient prognosis in prostate cancer.

## CONFLICT OF INTEREST

The authors declare that they have no conflict of interest.

## FUNDING INFORMATION

Cancer Prevention Research Institute of Texas (CPRIT); Grant Number: RR180061; National Cancer Institute of the National Institutes of Health; 1R21CA227996

## AUTHOR CONTRIBUTIONS

Chao Cheng conceived of the idea and designed data analysis. Emily Zhou and Baoyi Zhang performed the data analysis and prepared figures and tables. Emily Zhou wrote the manuscript with the help from Chao Cheng, Runjun D. Kumar, Evelien Schaafsma, Baoyi Zhang, and Kenneth Zhu. All authors discussed the results and contributed to the final manuscript.

## Supporting information

Appendix

FigureS1

FigureS2

FigureS3

TableS1

TableS2

TableS3

## Data Availability

The datasets supporting the conclusions of this article are available in the Gene Expression Omnibus repository (GSE16560 and GSE8402).
