# Supplementary material for: A TMPRSS2‐ERG gene signature predicts prognosis of patients with prostate adenocarcinoma
Source: Clin Transl Med. 2020 Dec 2;10(8):e216. doi: 10.1002/ctm2.216 (PMC7711082; doi:10.1002/ctm2.216)
Supplement: Supplementary file 1 — Appendix [file CTM2-10-e216-s001.docx]

**Supplemental Method**

**Prostate adenocarcinoma dataset**

The Cancer Genome Atlas (TCGA) prostate adenocarcinoma (PRAD) RNA-seq data was used to generate the TMPRSS2-ERG gene signature. The processed TCGA-PRAD data was downloaded from Firehose (http://gdac.broadinstitute.org). The TCGA-PRAD dataset contains the expression profile for 497 prostate tumor samples with RSEM-normalized counts for 20,502 gene. Gene fusion information was downloaded from TumorFusions ([https://www.tumorfusions.org/](about:blank)). The immune information for leukocyte abundance was downloaded from the Thorsson et al. study [1]. Immune infiltration for the six immune cells (Naïve B-cell, Memory B-cell, CD8^+^ T-cell, CD4^+^ T-cell, Natural Killer cell, and monocytes) were inferred using the computation method from the Varn et al. study [2].

Additional microarray-generated prostate cancer expression datasets were used in this study to verify the accuracy of the TMPRSS2-ERG gene signature. The two datasets used in this study can be found in the Gene Expression Omnibus (GEO) database with the following GEO accession IDs: GSE16560 [3] and GSE8402 [4], respectively. Additional information on these two datasets can be found in Supplementary Table 1.

**Define the TMPRSS2-ERG gene signature**

We defined a TMPRSS2-ERG gene signature that recapitulates downstream activity in the fusion pathway by comparing differential gene expression of fusion-positive and fusion-negative prostate cancer samples. The analysis was performed on the TCGA-PRAD RNA-seq dataset. The data was initially transformed using log(RPKM+1) to avoid extreme values. Then, we performed a logistic regression on each gene. The predictor variables were gene expression, age, and Gleason score. The response variable was fusion status. Using this model, we determined the β- and p-values for each gene. Weight profiles were assigned based on the following expressions: w^+^ = -log(p-value)I(β-value > 0) and w^_^ = -log(p-value)I(β-value < 0). We adjusted the weight profiles to fit into the [-1, 1] range. Genes were determined to be up-regulated when w^+^ is positive, and down-regulated when w^-^ is negative. The 100 most significant genes were chosen to become the TMPRSS2-ERG gene signature.

**Calculation of sample-specific TMPRSS2-ERG signature score based on expression profile**

We utilized the statistical method BASE [5, 6] on all three prostate adenocarcinoma datasets to calculate the sample-specific TMPRSS2-ERG signature scores (signature score). BASE uses a rank-based algorithm to determine the similarity between the TMPRSS2-ERG gene signature and the gene expression profiles of prostate tumor samples. Specifically, BASE begins by performing median normalization across samples on each gene to find relative gene expression levels. The relative gene expression profile is then constructed by ordering the normalized gene expression data in decreasing order. Then, the relative gene expression profile is analyzed with a foreground and background function [6]. The difference between the foreground and background function was normalized with a null distribution found with permutation. The maximum difference between the foreground and background function was labeled as fusion^+^ and the minimum difference was labelled as fusion^-^. We combined these two scores together (fusion^+^ - fusion^-^) to determine the sample-specific signature score. Higher signature scores are associated with a higher likelihood of TMPRSS2-ERG fusion in the prostate cancer sample.

**Predict patient survival using TMPRSS2-ERG signature score**

We performed survival analysis with the R library “survival”. Using the median as the threshold, signature score was dichotomized into two equally sized groups: high and low score. Cox proportional hazard models were used to determine the association between TMPRSS2-ERG signature score and patient survival. Multivariate Cox regression models were used to find the relationship between signature score and patient survival while adjusting for confounding variables. The confounding variables include: age, Gleason score, and extreme (indolent or lethal). We used the Kaplan-Meier method to plot the survival curves. Log-rank tests were performed to assess the difference between survival curves and their statistical significance.

**Supplemental Table**

**Supplemental Table 1: Prostate adenocarcinoma microarray datasets used in this study.**

For each dataset, its GEO ID, platform, author, PMID, survival information type, number of samples, number of fusion-positive and fusion-negative, age, Gleason score, and indolent and lethal tumor information. Some information was missing from the data in the Sboner (GSE16560) dataset.

**Supplemental Table 2: Signature score correlated with immune cell infiltration in TCGA-PRAD samples.**

The association of Naïve B-cell, Memory B-cell, CD8^+^ T-cell, CD4^+^ T-cell, Natural Killer cell, and monocyte infiltration with TMPRSS2-ERG signature score was analyzed. Natural Killer cells and monocytes infiltration are inversely correlated with signature score. CD4^+^ T-cells are positively correlated with signature score. Correlation and p-values were calculated using Spearman correlation analysis.

**Supplemental Table 3: GO enrichment analysis of signature genes**

Signature genes are enriched in hormone-related biological processes.

**Supplemental Figure**

**Supplemental Figure 1**

**A-B.** Fusion-positive samples have significantly higher signature scores in both TCGA-PRAD and Sboner (GSE16560) datasets (Wilcoxon test). The number of samples in each group can be found within the parenthesis. **C-D.** In both Sboner (GSE16560) and Setlur (GSE8402) dataset, fusion-positive samples have significantly higher ERG expression (Wilcoxon test).

**Supplemental Figure 2**

**A.** The sample-specific signature scores were dichotomized into two groups using the median as the threshold. Patients with high signature scores have significantly poorer prognosis than patients with low signature scores (Log-rank test). Number of patients in each sample is shown in the parenthesis. **B.** The ERG expression level of prostate tumor samples was dichotomized based on median ERG expression. Samples with high ERG expression levels have worse prognosis (Log-rank test). **C.** Fusion-positive samples with low ERG expression levels have significantly worse prognosis than fusion-positive samples with high ERG expression levels (Log-rank test). **D.** Fusion-negative samples with high ERG expression have similar prognosis compared to fusion-negative samples with low ERG expression (Log-rank test).

**Supplemental Figure 3**

**A.** A comparison of signature score among Gleason grades. All analyses are performed using the Sboner (GSE16560) dataset. **B.** Signature score is predictive of prognosis in fusion-negative Gleason 7 (G7) patients. Patients with high signature scores have significantly worse prognosis (Log-rank test). Number of patients in each group is shown in the parenthesis. **C.** Signature score can differentiate between lethal and indolent tumor samples. Lethal tumor samples are from patients who either died of prostate cancer or the prostate cancer metastasized. Indolent tumor samples are from patients who survived more than 10 years without metastasis. Patients in the lethal group have significantly higher signature scores compared to their counterparts (Wilcoxon test). Number of patients in each group is shown in the parenthesis. **D.** Lethal tumor samples have significantly higher signature scores in fusion-negative samples (Wilcoxon test). **E.** Lethal tumor samples have significantly higher signature scores in Gleason 7 (G7) samples (Wilcoxon test). **F.** Lethal tumor samples have significantly higher signature scores in Gleason 7 & fusion-negative samples (Wilcoxon test).

**References for Supplemental Material:**

1. Thorsson V, Gibbs DL, Brown SD, et al (4) The Immune Landscape of Cancer. Immunity 48:812-830.e14. https://doi.org/10.1016/j.immuni.2018.03.023

2. Varn FS, Wang Y, Mullins DW, et al (3) Systematic Pan-Cancer Analysis Reveals Immune Cell Interactions in the Tumor Microenvironment. Cancer Res 77:1271–1282. https://doi.org/10.1158/0008-5472.CAN-16-2490

3. Sboner A, Demichelis F, Calza S, et al (2010) Molecular sampling of prostate cancer: a dilemma for predicting disease progression. BMC Med Genomics 3:8. https://doi.org/10.1186/1755-8794-3-8

4. Setlur SR, Mertz KD, Hoshida Y, et al (2008) Estrogen-dependent signaling in a molecularly distinct subclass of aggressive prostate cancer. J Natl Cancer Inst 100:815–25. https://doi.org/10.1093/jnci/djn150

5. Cheng C, Yan X, Sun F, Li LM (2007) Inferring activity changes of transcription factors by binding association with sorted expression profiles. BMC Bioinformatics 8:452. https://doi.org/10.1186/1471-2105-8-452

6. Zhao Y, Varn FS, Cai G, et al (1) A P53-Deficiency Gene Signature Predicts Recurrence Risk of Patients with Early-Stage Lung Adenocarcinoma. Cancer Epidemiol Biomarkers Prev 27:86–95. https://doi.org/10.1158/1055-9965.EPI-17-0478
