# Supplementary figures and images for: A TMPRSS2‐ERG gene signature predicts prognosis of patients with prostate adenocarcinoma
Source: Clin Transl Med. 2020 Dec 2;10(8):e216. doi: 10.1002/ctm2.216 (PMC7711082; doi:10.1002/ctm2.216)

**A****TCGA**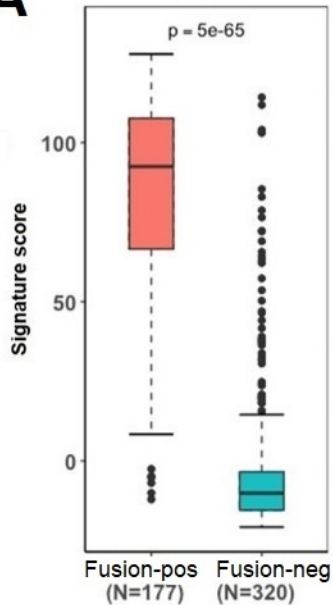**B****Sboner**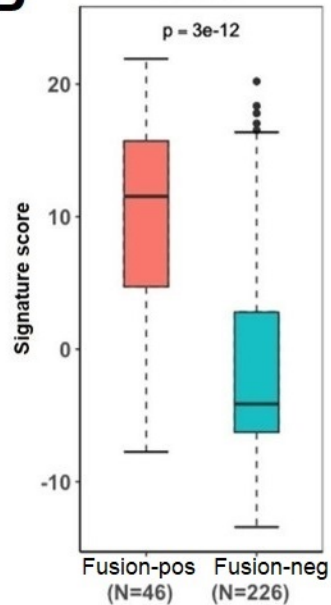**C****Sboner**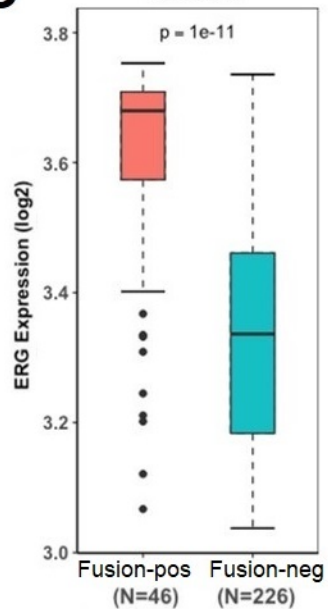**D****Setlur**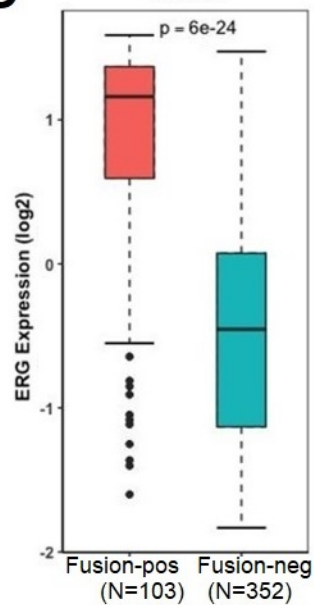

Supplement: Supplementary file 2 — FigureS1 [file CTM2-10-e216-s002.pdf]

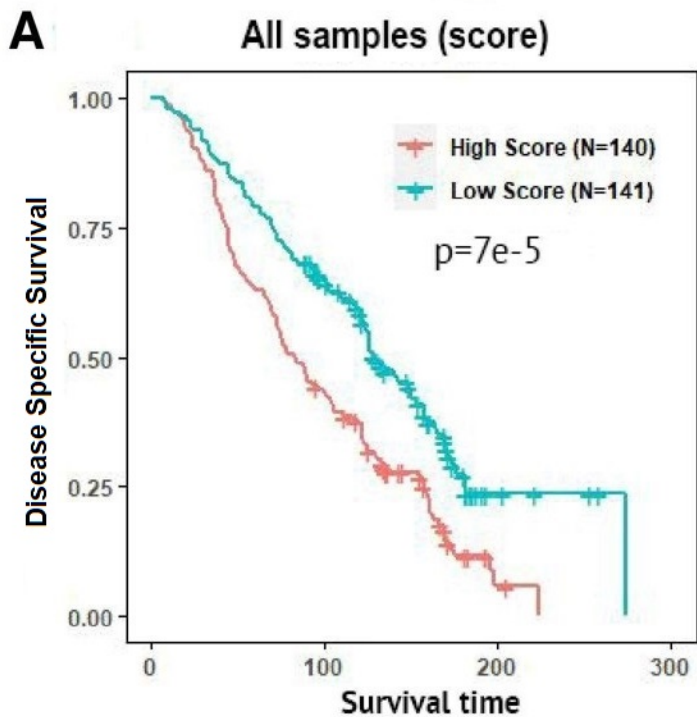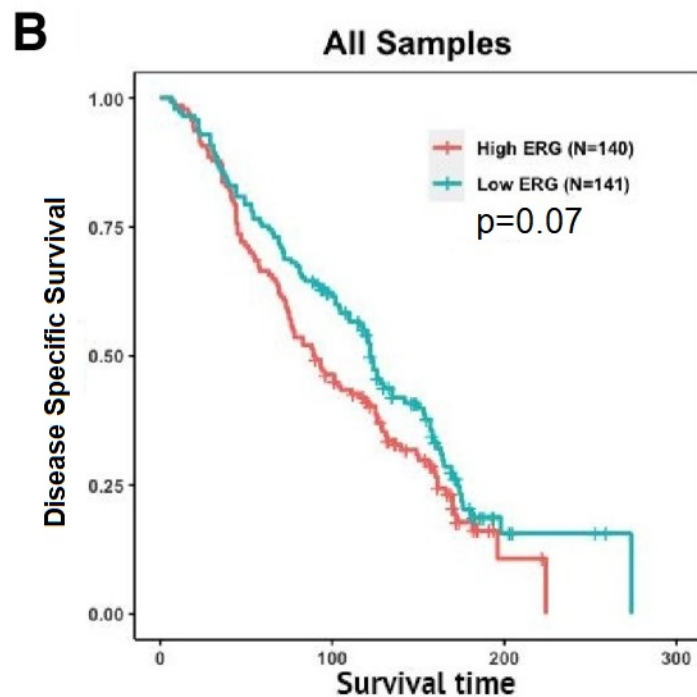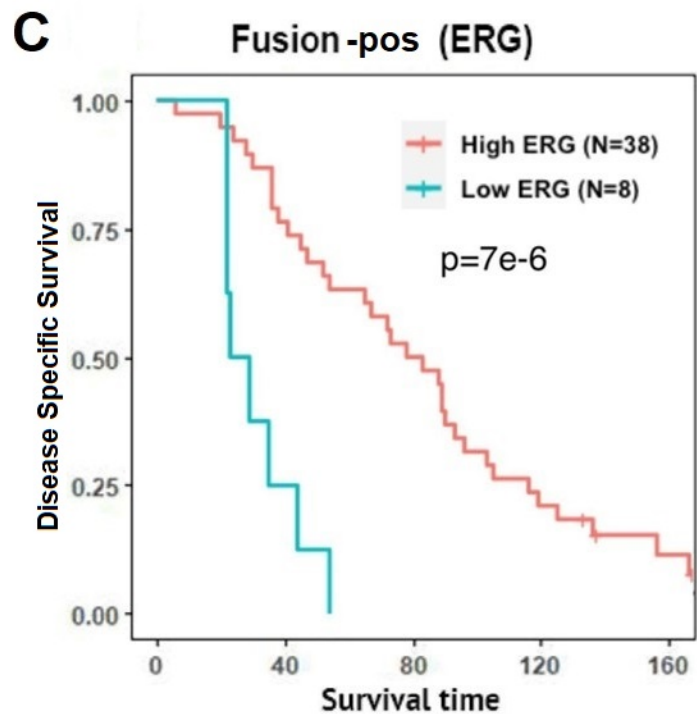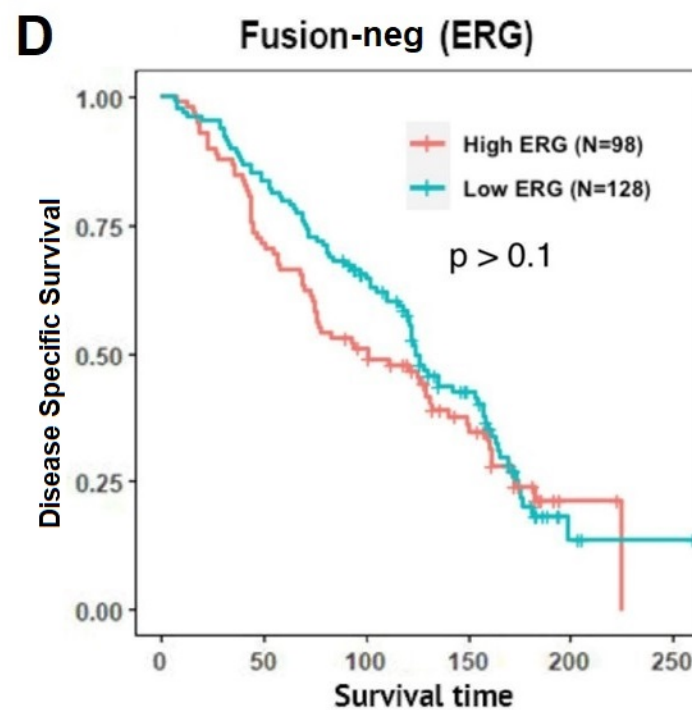

Supplement: Supplementary file 3 — FigureS2 [file CTM2-10-e216-s003.pdf]

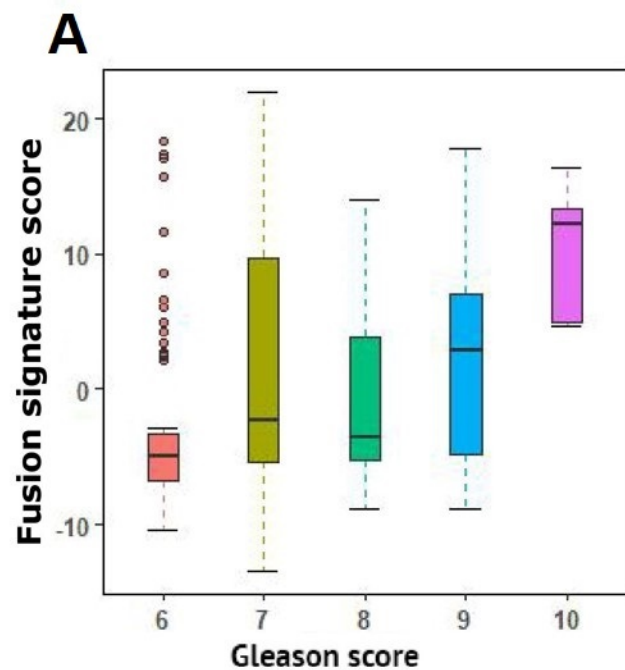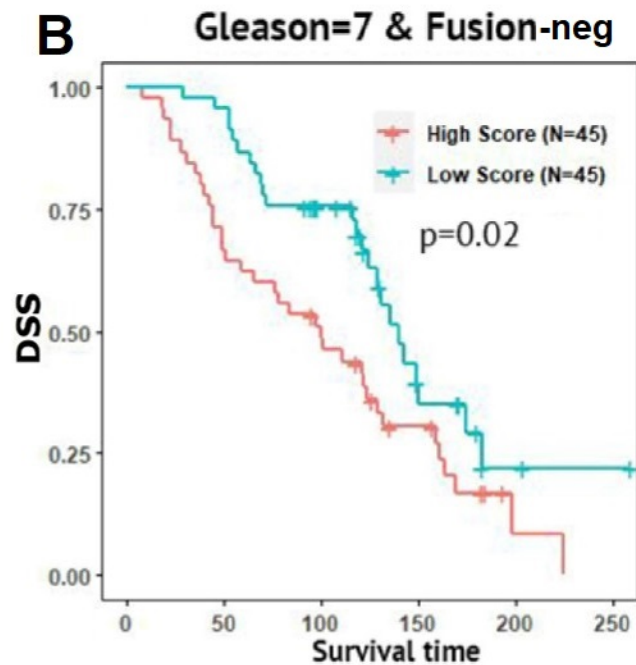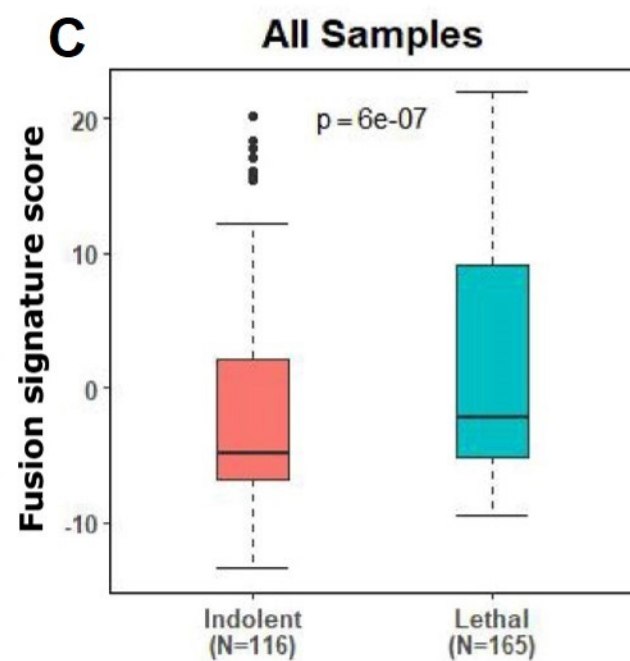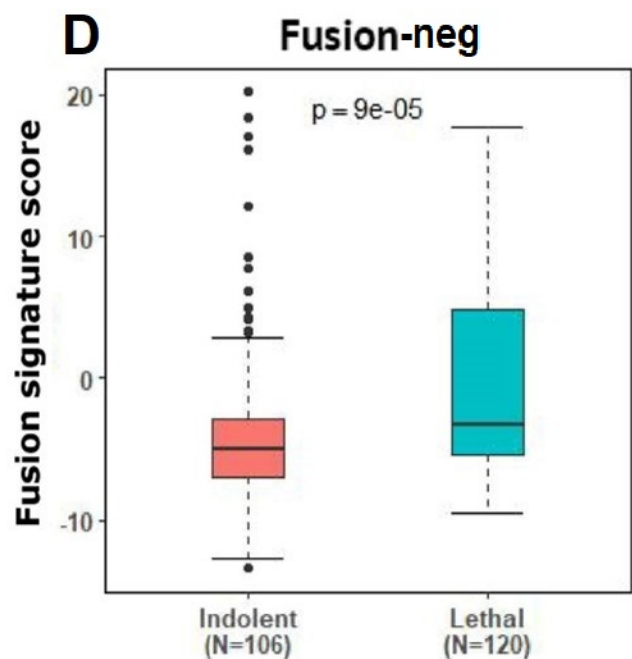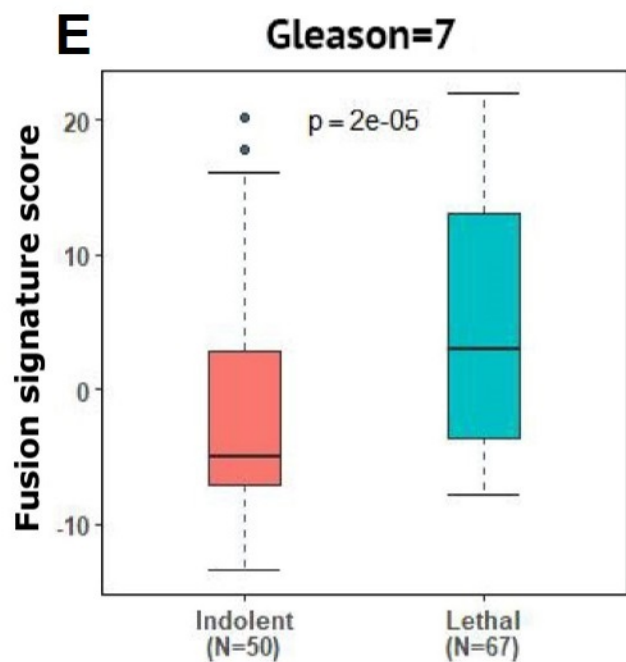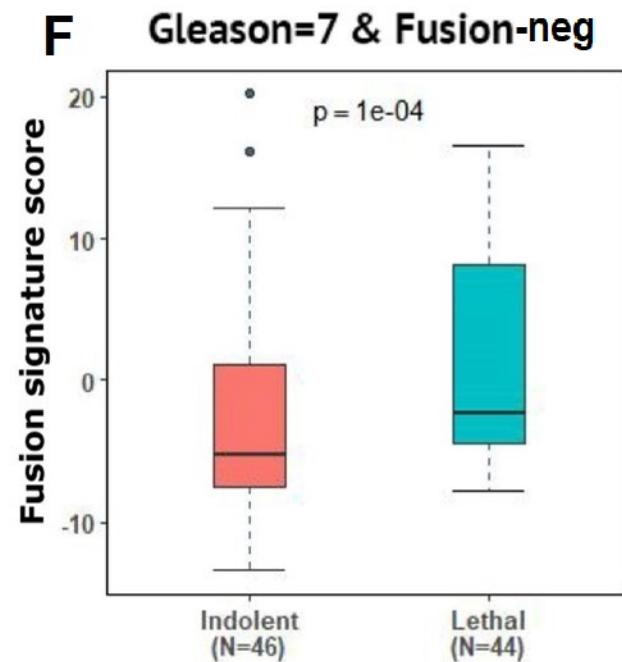

Supplement: Supplementary file 4 — FigureS3 [file CTM2-10-e216-s004.pdf]
